# Ultrasound Diagnosis of Clavicle Fractures in Newborns: A Systematic Review

**DOI:** 10.3390/children11091080

**Published:** 2024-09-03

**Authors:** Luca Galimberti, Gisella Garbetta, Antonella Poloniato, Rosanna Rovelli, Graziano Barera, Nicola Guindani, Maurizio De Pellegrin

**Affiliations:** 1 Department of Pediatrics and Neonatology, San Raffaele Hospital, 20132 Milan, Italy; garbetta.gisella@hsr.it (G.G.); poloniato.antonella@hsr.it (A.P.); rovelli.rosanna@hsr.it (R.R.); barera.graziano@hsr.it (G.B.); 2 Pediatrics Residency, Vita-Salute San Raffaele University, 20132 Milan, Italy; 3 Department of Orthopedics, Regional Health Care and Social Agency, Papa Giovanni XXIII Hospital, 24127 Bergamo, Italy; nicola.guindani@unimib.it (N.G.); depellegrin1956@gmail.com (M.D.P.); 4 Pediatric Orthopedic Unit, Piccole Figlie Hospital, 43125 Parma, Italy

**Keywords:** clavicle fractures, newborns, neonatal injuries, birth injuries, sonography, ultrasound, ultrasound diagnosis

## Abstract

Background: Fractures of the clavicle are the most common birth injury among newborns. Aim of this systematic review was to provide a comprehensive analysis of the role of ultrasound (US) in diagnosing clavicular fractures in neonates. Methods: A systematic review was conducted according to the Preferred Reporting Items for Systematic Reviews and Meta-Analyses (PRISMA) using PubMed and Embase, including studies focusing on US in neonatal clavicle fracture. Age at US, number of cases examined by US and X-ray, US and X-ray diagnoses, US probe used, fracture site were systematically extracted. Results: A total of 231 articles were found. We ultimately selected 7 publications that satisfied the inclusion criteria, involving 136 patients examined between 3 days and 3 weeks of age, with 135 confirmed fractures. US was performed on all patients and correctly diagnosed all fractures (135/135, 100%). X-ray was performed on 94/136 patients (69.1%) and correctly diagnosed 89/93 fractures (95.7%). Fracture site was: medial in 2/79, middle in 37/79, and lateral in 40/79. In the remaining 57 cases, site was not reported. Conclusions: This review indicates that ultrasound is extremely reliable in diagnosing clavicle fractures in newborns and should be considered as the gold standard in this context.

## 1. Introduction

Fractures of the clavicle are the most common birth injury, with reported incidence rates ranging from 0.5% to 4% among newborns [1,2,3,4,5,6,7,8,9]. Frequently cited risk factors include high birth weight or length, shoulder dystocia, prolonged labor, and vacuum-assisted delivery. Although less common, clavicle fractures have also been described in cesarean deliveries [4,6,7,9].

Neonatal clavicular fractures typically resolve spontaneously without long-term complications. However, proper diagnosis and follow-up are essential to assess complete healing and reassure parents, who can find the event worrisome in their newly born infant. Therefore, the diagnostic method used to confirm clinical suspicion should be reliable, easy to perform, and universally reproducible.

Most clavicle fractures in neonates are of the “greenstick” type, thus being less apparent during clinical examination. Clinical signs associated with this type of fracture have been recognized for decades, with the most common being the presence of a palpable mass, asymmetry of spontaneous movements or Moro reflex, bone profile irregularity, and pain upon palpation [2,3].

The interest in ultrasound (US) for diagnosing pediatric fractures has grown over the last decades. Numerous studies regarding a large number of patients have consistently shown its reliability, with better results compared to X-ray in some cases. Several studies have focused on the use of sonography in the setting of the emergency department. The first study to do so, to our knowledge, was published in 1999 by Blab et al. and showed no statistical differences between US and X-ray for pediatric clavicle fractures [10]. Subsequent studies have confirmed these results in children of any age [11,12,13,14,15]. Most recently, the Australian work group of Snelling et al. conducted a multicenter randomized controlled trial focused on forearm fractures among 270 children and proved the noninferiority of US compared to X-ray in this setting, confirming the widespread interest for this technique [16,17].

Data regarding sonographic diagnosis of all-age clavicle fractures are abundant and confirm its reliability. A recent systematic review [18] considered relevant papers on adult and pediatric populations and concluded that the currently available literature supports US as a reliable imaging tool for the diagnosis of clavicle fractures, with significantly high sensitivity and specificity (94% and 98%, respectively).

Unfortunately, only a few studies have been directed at the neonatal population exclusively. Traditionally, the diagnosis of neonatal clavicular fracture has relied heavily on conventional X-ray imaging, which exposes neonates to ionizing radiation. Sonographic diagnosis in this setting is not yet widespread, and conventional X-ray imaging is still widely used as the routine diagnostic method [9,19,20]. Furthermore, there is a scarcity of guidelines from international societies on the matter.

The aim of this systematic review is to provide a comprehensive analysis of the role of US in the diagnosis of clavicular fractures in neonates.

## 2. Materials and Methods

### 2.1. Database Sources and Search Strategies

This systematic review was conducted by a single author according to the guidelines of the Preferred Reporting Items for Systematic Reviews and Meta-Analyses (PRISMA) on 27 June 2024 using two medical electronic databases (PubMed and Embase). The research string used was: (neonatal OR newborn OR infant) AND clavic* AND (sonograph* OR ultrasound OR echograph*) AND fracture. In total, 231 articles were found: 155 and 76 in Pubmed and Embase, respectively.

### 2.2. Article Eligibility Criteria

The initial titles and abstracts were screened using the following inclusion criteria: studies of any level of evidence reporting data on ultrasound in the setting of clavicle fracture in infants. All articles that dealt with different topics or were without an accessible abstract were excluded.

The following exclusion criteria were adopted for the papers that met the inclusion criteria after full-text reading: papers not focusing on the neonatal age, not focusing on fractures of the clavicle or not providing precise data regarding the cases.

### 2.3. Data Collection

From each article, individual patient data were extracted, and the following information was gathered:

- Authors and publication date
- Study type
- US probe used
- Sonographic signs of fracture
- Age at US
- Number of cases examined by US
- Number of cases examined by X-ray
- Number of fractures diagnosed by US
- Number of fractures diagnosed by X-ray
- Fracture localization (medial, middle or lateral third)

Number of examined patients, number of US and X-ray performed and number of diagnoses of fracture were extrapolated from each study and expressed as fractions and percentages.

The collection of data was executed utilizing Microsoft Excel (Version 2024, Microsoft Corporation, Redmond, WA, USA).

## 3. Results

**Figure 1:**
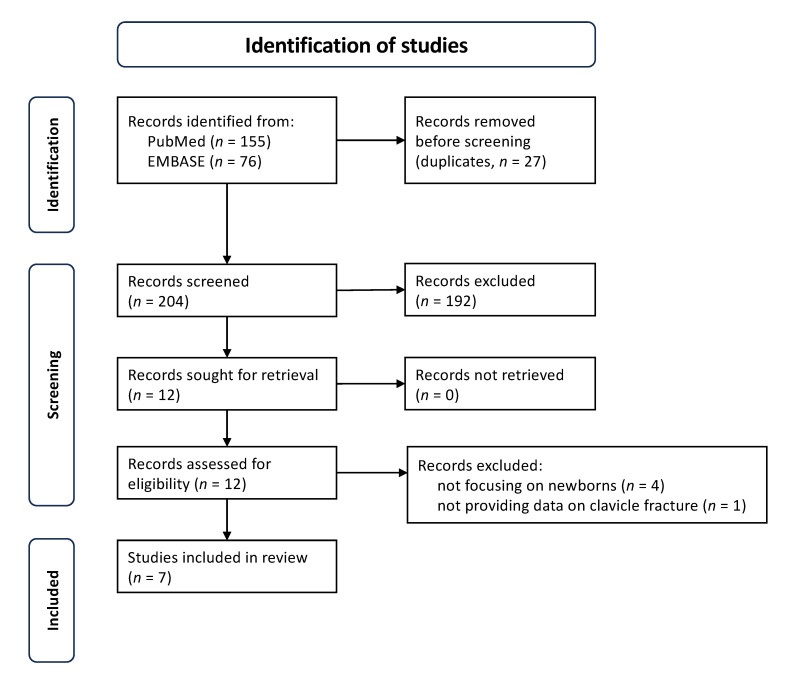
PRISMA flowchart for the selection and screening method for relevant publications.

After the systematic review of literature, a total of 231 articles were found. Following the removal of duplicates and screening by title, abstract and full-text, at the end of the initial screening, 12 publications were deemed eligible for inclusion and for reading in full text [10,13,14,15,21,22,23,24,25,26,27,28]. After full-text reading, we ultimately selected 7 publications that satisfied the inclusion criteria [21,22,23,24,25,26,27]. A PRISMA flowchart of the selection and screening method is provided in Figure 1.

### 3.1. Study Characteristics

Among the 7 included studies, a total of 136 newborns presented with suspected clavicle fractures. The characteristics of the included studies are described in Table 1. Five case series and two case reports were considered.

All 136 newborns underwent ultrasound examination. In all but three studies, all patients were also examined using X-ray; in the two case series by Blankstein et al. [27] and Liu et al. [23], radiography was only performed in 47.6% and 65.2% of the cohort, respectively; in the remaining study, no radiograph was performed. In the latter study, from 2003, Kayser and colleagues [25] evaluated 2978 newborns and identified a clinical suspicion of clavicle fracture in 15 of them: ultrasound confirmed the diagnosis in all 15. The working group didn’t perform radiographs, citing the previous work from Katz et al. [21] and suggesting that ultrasound alone, in combination with clinical suspicion, is a sufficient method for typical clavicle fractures; though, they recommended performing radiography in cases of neurological injury and for congenital pseudarthrosis. In total, 94/136 patients underwent radiographic examination (69.1%).

The presence of a fracture was confirmed by either sonography or X-ray in 135 out of 136 cases; in the remaining case, no fracture was detected by neither sonography nor X-ray, thus confuting the clinical suspicion [22].

The site of the fracture was reported by the studies for 79 out of the 135 fractures. The lateral third was the most common fracture site (50.6%), followed by the middle third (46.8%) and the median third (2.6%).

### 3.2. Criteria for Cinical and Sonographic Diagnosis

For most of the examined papers, clinical suspicion of clavicle fracture was particularly based on two signs upon examination: presence of a palpable spongy mass [21,23] and irregularity of spontaneous movements [21,23,26].

Ultrasound examination was performed in all cases with the baby in a supine position, examining both clavicles, with the infant’s head rotated contralaterally to the examined side. A linear, high-frequency probe was used in all cases. Depending on the studies, US was performed within the first days of life or by the age of three weeks.

The most common sonographic sign of a fracture is cortical bone interruption, particularly visible on a longitudinal scan. Periosteal lesions and soft tissue hematomas are also frequently observed [21,22,23,24,25,26,27]. In more severe cases involving complete fractures, dynamic sonographic examination may reveal dislocation or movement of the bone fragments during respiration [21,22,23,27]. In some studies, patients were monitored with follow-up sonographic examinations, which demonstrated the formation and subsequent resolution of a perilesional callus [21,22,23,25,27].

### 3.3. Performance of the Imaging Methods

The combined data from the identified studies enable an assessment of the reliability of the two diagnostic methods in accurately diagnosing clavicular fractures. A definitive diagnosis of fracture was based on the presence of clinical suspicion associated with characteristic signs observed on either imaging method.

Sonography correctly diagnosed 135/135 fractures (100%). Among the patients that underwent X-ray as well, 89/93 fractures were identified (95.7%). Data from the specific studies are reported in Table 1.

## 4. Discussion

For the diagnosis and management of clavicle fractures in newborns, conventional X-ray imaging remains the routine diagnostic method, while US is not yet widely adopted. The main finding of this systematic review, which included 136 patients with 135 confirmed fractures, is that ultrasound is a reliable and radiation-free alternative to conventional X-ray imaging for diagnosing clavicular fractures in newborns

Data available from the literature indicate that interest in using US for diagnosing neonatal clavicle fractures emerged independently in two separate working groups around the same time. The first two papers on this topic were published by Katz et al. in 1988 [21] and by Bartoli et al. in 1989 [22]. Unaware of each other’s work, both groups examined a series of newborn infants using similar methodologies and successfully identified fractures using sonography in all cases. Both teams then compared the sonographic images with plain radiographs, demonstrating that ultrasound was noninferior to X-ray; notably, in the case series by Katz et al., one fracture was not visible on radiography. Based on these findings, both studies suggested that ultrasound should be the only diagnostic method to confirm clinical suspicion of a clavicular fracture in newborns. It is particularly noteworthy that these pioneering case series were published over 35 years ago, during a time when ultrasound imaging was still seminal and only able to provide low-resolution images. Subsequent research, although limited in number, has consistently validated these results. Over this period, very few research papers investigated this matter, but consistently proved the same results. Specifically, the recent work by Liu and colleagues, which was designed to assess the sensitivity and specificity of ultrasound, confirmed its accuracy in a rigorous manner [23].

In two of the five case series considered, sonography successfully identified a fracture that was missed by X-ray, demonstrating higher sensitivity than the imaging modality currently considered the gold standard. In the seminal study by Katz et al. [21], a fracture in the medial third of the clavicle was not detected on the initial chest radiograph but became visible on a subsequent X-ray in the lordotic position after the ultrasound diagnosis. Similarly, in the recent study by Liu et al. [23], an X-ray performed due to postnatal dyspnea failed to detect a middle third fracture; the subsequent physical examination raised the clinical suspicion of fracture, which was confirmed by ultrasound.

Additionally, both case reports that we identified focused on fractures missed by X-ray. In the case reported by Drakonaki et al. [26], a middle third fracture was not identified by radiography in a newborn presenting with upper limb pseudoparalysis, whereas sonography revealed interruption of the cortex and an associated overlying hypoechoic hematoma. The case presented by Baessler et al. [24] involved a displaced physeal fracture initially misinterpreted as a left sternoclavicular dislocation on X-ray in an infant also presenting with upper limb pseudoparalysis.

False negatives in this context can occur due to the neonate’s positioning, and consequently the position of the clavicles, during X-ray imaging; the fact that the radiographs were not specifically focused on the clavicles; and the clavicle’s anatomy, which physiologically shows overlapping parts due to its curvature in the middle region. One of the key advantages of ultrasound is its ability to follow the entire bone cortex and detect secondary signs of fracture, such as subperiosteal hematoma.

In the studies examined, the sensibility of ultrasound was found to be extremely high, with no additional fractures detected by X-ray in the patients who underwent both ultrasound and radiography [21,22,23,24,26,27].

Sonographic examination of a superficial bone like the clavicle is an easy-to-learn technique that can be performed by the clinician at the newborn’s bedside, allowing for a timely and convenient diagnosis. Due to the clavicle’s superficial position, evaluation via high-frequency linear probes provides high-resolution images of the entire bone, allowing for the assessment of direct and indirect signs of fracture. Cortical bone interruption is the most important sign to identify. The current widespread availability of sonographs can be particularly beneficial for diagnosing neonatal clavicle fractures in low- and middle-income countries, where more expensive imaging tools are scarce, especially in small, peripheral hospitals. Additionally, because ultrasound does not involve ionizing radiation, it can be used repeatedly to monitor bone healing over time [10]. Even though clavicle fractures at this age typically do not require intervention, ongoing monitoring can reassure parents who may be alarmed by this diagnosis, even in an otherwise healthy newborn.

The findings of this review support the broader adoption of ultrasound in clinical practice for this purpose. Achieving this in the future will require enhancing training programs for healthcare providers and conducting additional large, prospective studies to gather more data, possibly including children without clinical signs of fracture. Currently, recommendations from international scientific societies are lacking on this matter, and such guidelines would be crucial in guiding practitioners toward adopting ultrasound as a routine diagnostic tool.

### Study Limitations

We acknowledge that the number of studies and the total number of patients considered in this review is relatively small, owing to the limited research interest on this specific topic.

Additionally, in all the considered studies, imaging—either ultrasound or radiography—was only realized following the clinical suspicion of fracture, thus possibly over-estimating the accuracy of the diagnostic imaging modalities.

## 5. Conclusions

This systematic review confirms that US is an extremely reliable tool for diagnosing clavicle fracture in newborns. The widespread adoption of US as the gold standard in this context should be recommended.

## Figures and Tables

**Table 1:** Characteristics of studies and patients enrolled in the reviewed studies. US, ultrasound; n/a, not available; NICU, neonatal intensive care unit.

Author	Katz	Bartoli	Blankstein	Kayser	Drakonaki	Baessler	Liu	Totals
**Year**	1988 [21]	1989 [22]	2001 [27]	2003 [25]	2010 [26]	2020 [24]	2022 [23]	
**Study type**	Case series	Case series	Case series	Case series	Case report	Case report	Case series	
**Probe type**	Linear	Linear	Linear	Linear	n/a	n/a	Linear	
**Probe frequency**	6–10 MHz	5–7.5 MHz	6–13 MHz	7.5 MHz	n/a	n/a	4–12 MHz	
**Age at US**	First 3 days	Upon suspicion	First 3 weeks	3 days	3 days	12 days	NICU entry	
**Cases investigated**	41	11	21	15	1	1	46	136
**Cases examined by US**	41/41 (100%)	11/11 (100%)	21/21 (100%)	15/15 (100%)	1/1 (100%)	1/1 (100%)	46/46 (100%)	136/136 (100%)
**Cases examined by X-ray**	41/41 (100%)	11/11 (100%)	10/21 (47.6%)	0/15 (0%)	1/1 (100%)	1/1 (100%)	30/46 (65.2%)	94/136 (69.1%)
**Confirmed diagnoses**	41	10	21	15	1	1	46	135
**Fractures diagnosed by US**	41/41 (100%)	10/10 (100%)	21/21 (100%)	15/15 (100%)	1/1 (100%)	1/1 (100%)	46/46 (100%)	135/135 (100%)
**Fractures diagnosed by X-ray**	40/41 (97.6%)	10/10 (100%)	10/10 (100%)	n/a	0/1 (0%)	0/1 (0%)	29/30 (96.7%)	89/93 (95.7%)
**Fracture site**								
**Medial third**	1/41	n/a	0/21	0/15	0/1	1/1	n/a	2/79 (2.6%)
**Middle third**	14/41	n/a	7/21	15/15	1/1	0/1	n/a	37/79 (46.8%)
**Lateral third**	26/41	n/a	14/21	0/15	0/1	0/1	n/a	40/79 (50.6%)

## Data Availability

Data are contained within the article.
